# Use of tranexamic acid in trauma surgical specialties: a narrative review

**DOI:** 10.1186/s13017-025-00649-9

**Published:** 2025-10-10

**Authors:** Hannah M. Thomas, Huthayfa Kahf, Benjamin Bush, Jeffry Nahmias, Philip K. Lim

**Affiliations:** 1 Department of Orthopaedic Surgery, University of California, Irvine, 101 The City Drive South, Pavilion 3, Building 29A, Orange, CA 92868 USA; 2 Dodge Family College of Arts and Sciences, University of Oklahoma, 660 Parrington Oval, Norman, OK 73019 USA; 3 Department of Surgery, University of California, Irvine, 101 The City Drive South, Orange, CA 92868 USA

**Keywords:** Hemostasis, Coagulopathy, Tranexamic acid, Fibrinolysis, Trauma, Prehospital care, Massive transfusion, Hemorrhage, Resuscitation, Mortality

## Abstract

**Background:**

Tranexamic acid (TXA) is a well-known antifibrinolytic agent with increasing evidence supporting its use in trauma patients. This review evaluates the current available literature regarding TXA and its potential use to improve patient survival and reduce transfusion needs across multiple trauma surgical subspecialties and contexts.

**Methods:**

A literature review was conducted on the efficacy and safety of tranexamic acid in trauma surgical specialties using PubMed (MEDLINE) and Google Scholar from database inception to October 2024. Selected articles were written in the English language and encompassed reviews, experimental studies, and basic science articles.

**Results:**

There is conflicting evidence on the mortality benefit of TXA, particularly in the prehospital setting. However, multiple large, high-quality studies have shown that TXA is an effective agent to reduce bleeding after trauma. Extensive evidence exists that TXA is a safe medication, with numerous studies demonstrating no increased risk of thromboembolic events after administration of TXA in trauma settings. Additionally, multiple cost-effectiveness studies conducted in several countries have found TXA to be a highly cost-effective intervention following trauma.

**Conclusion:**

TXA is a safe, effective, and cost-effective medication to reduce bleeding after trauma. Future research on TXA is needed to elucidate the potential benefit of TXA after traumatic brain and spine injury and the optimal dose and route of administration of TXA.

## Background

Acute trauma is a leading cause of death in adults worldwide, with trauma-associated hemorrhage being a major contributor to morbidity and mortality in these patients [1, 2]. In addition, the concurrent development of coagulopathy secondary to trauma can further exacerbate hemorrhage due to hyperfibrinolysis, with some studies showing a mortality rate as high as 70–100% in traumatized patients in a hyperfibrinolytic state [3, 4]. Given these factors, there is increasing interest in modalities to reduce hemorrhage in trauma patients to decrease morbidity and improve patient survival.

In 2010, the landmark Clinical Randomization of an Antifibrinolytic in Significant Hemorrhage (CRASH)-2 trial demonstrated that empiric administration of tranexamic acid (TXA), an antifibrinolytic, resulted in a 1.5% absolute reduction in 28-day mortality among 20,211 patients with or at risk of significant bleeding [5]. Since then, numerous subsequent studies have similarly found that TXA is associated with decreased mortality and risk of bleeding after trauma. However, several more recent studies have found no significant reduction in mortality rates after TXA administration in the pre-hospital setting or in trauma patients with hyperfibrinolysis [6–8].

Given these conflicting findings, this review seeks to summarize the existing body of evidence regarding the efficacy, safety, and cost of TXA across multiple trauma contexts including in-hospital administration, pre-hospital administration, orthopaedic/spine trauma, and neurological trauma.

## Methods

This is a narrative, non-systematic review of the efficacy and safety of tranexamic acid in trauma surgical specialties. Relevant scientific articles were identified by searching the PubMed (MEDLINE) and Google Scholar databases. Articles written from database inception to October 2024 were eligible for inclusion. Relevant ongoing clinical trials were identified and analyzed from ClinicalTrials.gov. Keywords searched were trauma, tranexamic acid, TXA, and antifibrinolytic. To identify literature related to orthopaedic and neurological trauma, additional searches were conducted adding the terms fracture, orthopaedic, neurosurgery, spine, and traumatic brain injury. For each section of the review, all identified relevant articles were screened and assessed for methodological quality by two authors. In total, 106 full-text articles were reviewed and considered for inclusion. As the total number of studies written on TXA is too large to include in a single review paper, studies of the highest methodological quality were prioritized for inclusion. Meta-analyses and large randomized controlled trials were selected over cohort studies and case reports. Selected articles were written in the English language and encompassed reviews, experimental studies, and basic science articles. Each selected article was then reviewed, and results related to the efficacy and safety profile of TXA were extracted.

### Mechanism of action and pharmacology

Functional hemostasis depends on a delicate balance between the coagulation cascade, which creates hemostatic fibrin clots, and the fibrinolytic system, which degrades them [9]. Antifibrinolytics, like TXA, work by mitigating clot breakdown [10]. TXA competitively inhibits the lysine binding site on plasminogen, which disrupts the interaction between plasminogen and fibrin, preventing clot breakdown [10]. TXA has a half-life of approximately two hours and is excreted via urine [11, 12]. The fibrinolytic suppression effect of TXA occurs at plasma concentrations between approximately 5 and 15 mg/L and can last up to 8 h [13, 14].

During major trauma, significant blood loss can disrupt the hemostatic system by causing a hyperfibrinolytic state in which systemic plasmin activation results in rapid degradation of fibrin clots [9]. This makes TXA a particularly valuable drug in trauma contexts, as it can potentially mitigate the effects of hyperfibrinolysis, which occurs in 2–34% of severe traumas and is associated with an increased risk of mortality [15]. Hyperfibrinolysis is most commonly defined via viscoelastic testing, such as thrombelastography, with an estimated lysis at 30 min of 3.0% or greater [7].

In addition to degradation of fibrin, plasmin is also known to have immunomodulatory effects, including monocyte and complement activation, and promotion of monocyte, macrophage, and dendritic cell migration [16–21]. Results from animal studies suggest that TXA may possess anti-inflammatory properties when used in trauma settings [22, 23]. However, the 2020 Tranexamic Acid Mechanisms and Pharmacokinetics in Traumatic Injury (TAMPITI) randomized clinical trial (RCT) found that in humans there was no significant difference in immunomodulation as measured by HLA-DR receptor expression, myeloid cell expression, lymphoid cell expression, or classical complement activation between patients that received a 2 g bolus of TXA, a 4 g bolus of TXA, or a placebo [24].

### Formulation options—dosage, route, timing

TXA is available in intravenous (IV), intramuscular (IM), oral, and topical formulations. In the United States, there are only two Food and Drug Administration (FDA) approved indications for TXA [25]. IV TXA is approved for heavy bleeding in hemophilia patients during tooth extractions, and oral TXA is approved for menorrhagia [25]. IM and topical TXA are not FDA-approved in any contexts. Despite the narrow range of labeled indications, TXA has been studied in many additional clinical scenarios including major trauma, postpartum hemorrhage, cardiac surgery, liver surgery, and orthopaedic surgery [25, 26]. In trauma contexts, TXA is commonly used off-label based on the results of the CRASH-2 trial [5].

In the CRASH-2 trial, TXA was administered intravenously as a 1 g loading dose followed by a 1 g infusion over the next 8 h [5]. This regimen was selected as it allowed for a fixed dose that achieved therapeutic inhibition of fibrinolysis in larger patients while still being safe for smaller patients [5]. The authors determined that the fixed dose was preferable compared to weight-based dosing as it eliminated the need to weigh the patient in the emergency setting [5]. Many subsequent studies of TXA use the same 1 g loading dose as the CRASH-2 study, with more variable subsequent dosing of boluses and/or infusions [6, 27–30]. Other studies use weight-based dosing, most commonly 10–15 mg/kg administered as a bolus [31–34]. Currently, there is no established optimal dose of TXA; however, more recently, there has been increased attention towards a higher initial dose of 2 g [35].

### Safety and adverse effects

Treatment with TXA is generally well tolerated with an excellent safety profile. In the CRASH-2 trial, there was no significant difference in the rate of vascular occlusive events, including myocardial infarction, stroke, pulmonary embolism (PE), and deep vein thrombosis (DVT), between patients that received TXA and patients that did not receive TXA (1.7% versus 2.0% respectively, *p* = 0.084) [5]. Multiple subsequent high-quality studies have similarly found no significant difference in the rate of thromboembolic events, including a large multicenter trial by Rivas et al. that included over 1,300 patients [6, 30–32, 34, 36–38]. Relative contraindications to TXA include active thromboembolic disorder, consumptive coagulopathies, renal failure, and subarachnoid hemorrhage [39]. Reported adverse effects include nausea, vomiting, diarrhea, and visual disturbances [40].

### Cost effectiveness

Based on the results of the CRASH-2 trial, it is estimated that widespread TXA use could save 70,000–100,000 lives around the world, and in 2011 TXA was added to the World Health Organization (WHO) list of essential medicines. Therefore, the cost-effectiveness of TXA is an important factor to consider, as its cost may still be prohibitively expensive for routine administration in lower-income countries.

One study to evaluate the cost-effectiveness of TXA was performed by Guerriero et al. using CRASH-2 trial data. The authors calculated that administering TXA early (within 3 h) in trauma patients with significant blood loss saved an estimated 372, 315, and 755 life years (LYs) per 1000 trauma patients in Tanzania, India, and the United Kingdom (UK), respectively [41]. The estimated total cost of administering TXA per patient was $17.48 in Tanzania, $19.55 in India, and $30.83 in the UK, and the authors concluded that TXA is highly cost-effective in all three countries.

Similarly, Williams et al. evaluated the cost-effectiveness of TXA in patients with TBI using CRASH-3 trial data by comparing the cost per quality-adjusted life years (QALY) in the UK and Pakistan [42]. In the UK, the calculated cost of TXA administration (e.g. 2 g of TXA, saline, syringes/needles, nurse administration time) was £4,288/QALY compared to a pre-determined cost-effectiveness threshold of £20,000/QALY. In Pakistan, the calculated cost of TXA administration was US$24/QALY vs a pre-determined cost-effectiveness threshold of US$158/QALY [42]. Therefore, they determined that TXA was 98% and 99% cost-effective at the pre-established cost-effectiveness thresholds for the UK and Pakistan, respectively.

On a smaller scale, Hubble et al. calculated the cost-effectiveness of a statewide pre-hospital IV TXA protocol for trauma patients with suspected hemorrhage in North Carolina (population 10.5 million) [43]. They estimated an absolute mortality reduction of 3%, 4.8 LYs saved per year, and 191 total life-years gained at a cost of $1595 per LY saved in the first year of this protocol. They calculated the implementation and operational cost for the first year of a pre-hospital TXA protocol to be $305,122, which would be approximately six times less than the potential reduction in total annual hospitalization costs, which was calculated to be $1,828,072. Therefore, they believe that a pre-hospital TXA protocol would be significantly cost-effective for trauma patients with suspected bleeding in the state of North Carolina.

### In-hospital TXA use

Many existing studies evaluate the efficacy of TXA in the context of general surgery trauma. Table 1 summarizes key general trauma studies. The landmark CRASH-2 trial enrolled over 20,000 adult trauma patients across 274 hospitals in 40 countries. Patients were given TXA for extracranial hemorrhage with vital signs including systolic blood pressure < 90 or heart rate > 110 beats per minute. The study found a lower all-cause 28-day mortality in the group receiving 1 g of TXA followed by 1 g infusion over 8 h compared to the control group (14.5% vs 16%, *p* = 0.0035). Interestingly, blood transfusion rates were similar between the TXA and control cohorts (50.4% vs. 51.3%, *p* = 0.21) [5].

**Table 1:** Key studies on the use of TXA in general surgery trauma contexts

Author name, year	Study design	Sample size	Key findings on TXA efficacy	Key findings on TXA safety
CRASH-2 Trial Collaborators [5]	Randomized controlled trial	20,211	All-cause 28-day mortality was significantly lower in the TXA group than the control group (14.5% vs. 16.0%, RR = 0.91, *p* = 0.0035)	There was no significant difference in the rate of thromboembolic events between the TXA and control groups (1.7% vs. 2.0%, RR = 0.84, *p* = 0.084)
Morrison et al. [27]	Retrospective cohort	896	In-hospital mortality was significantly lower in the TXA group than the control group (17.4% vs. 23.9%, *p* = 0.03). The mortality benefit was greatest among patients who required a massive transfusion protocol, (14.4% vs. 28.1%, *p* = 0.004)	The TXA group had a significantly higher risk of both pulmonary embolism (2.7% vs. 0.3%, *p* = 0.001) and deep vein thrombosis (2.4% vs. 0.2%, *p* = 0.001) compared to the control group. However, the TXA group also had higher ISS which is associated with higher rates of thromboembolic events
Morrison et al. [28]	Retrospective cohort	1,332	Both TXA and cryoprecipitate were associated with decreased in-hospital mortality (11.6% TXA + cryoprecipitate, 18.2% TXA only, 21.4% cryoprecipitate only, 23.6% neither TXA or cryoprecipitate). Compared to the control group, the TXA group had significantly reduced odds of mortality (OR = 0.61, *p* = 0.01)	No data on thromboembolic complications was reported
Valle et al. [8]	Case–control	300	In-hospital mortality was higher in the TXA group compared to the control group (27% vs. 17%, *p* = 0.024)	No data on thromboembolic complications was reported
Harvin et al. [7]	Retrospective cohort	1,032	In-hospital mortality was higher in the TXA group than the control group (40% vs. 17%, *p* < 0.001)	There was no significant different in the incidence of venous thromboembolism (6.3% vs. 4.4%, *p* = 0.389)
Guyette et al. [6]	Randomized controlled trial	927	There was no significant difference in mortality at 30 days between the TXA and control groups (8.1% vs. 9.9%, *p* = 0.17). However, in subgroup analysis, there was a mortality benefit among patients who received TXA within 1 h of injury (4.6% vs. 7.6%, *p* < 0.001)	There was no significant difference in the incidence of pulmonary embolism (2.9% vs. 1.5%, *p* = 0.78), DVT (2.7% vs. 1.5%, *p* = 0.83), or seizures (1.1% vs. 1.5%, *p* = 0.94) across groups
PATCH-Trauma Investigators [37]	Randomized controlled trial	1,310	Mortality at 24 h was lower among patients receiving TXA compared to placebo (9.7% vs. 14.1%, RR = 0.69, 95% CI 0.51 to 0.94), but survival at 6 months was not significantly different between the two groups (53.7% vs. 53.5%, RR = 1.00, *p* = 0.95)	The number of serious adverse events, including thromboembolic complications, did not differ significantly between the groups

Following the publication of CRASH-2, many additional studies were undertaken to explore the safety and efficacy of TXA after major trauma. The Military Application of TXA in Trauma Emergency Resuscitation (MATTERs) retrospective study found that the mortality benefit of TXA was greatest among patients who required a massive transfusion protocol (unadjusted mortality rate TXA vs no-TXA, 17.4% vs 23.9%, respectively; *P* = 0.03) [27]. Notably, the TXA group had a significantly increased risk of both pulmonary embolism (TXA vs no-TXA, 2.7% vs. 0.3%, respectively, *p* = 0.001) and deep vein thrombosis (2.4% vs. 0.2%, *p* = 0.001). However, they attributed this to the TXA group having a higher average Injury Severity Score (ISS), which at baseline is associated with higher rates of thromboembolic events [27]. The subsequent MATTERs II study expanded the scope of MATTERs I by evaluating the impact of TXA and cryoprecipitate (CRYO) on mortality [28]. Despite greater average ISS and transfusion requirements, in-hospital mortality was lowest in the TXA/CRYO (11.6%) and TXA only (18.2%) groups compared with the CRYO only (21.4%) and no TXA/CRYO (23.6%) groups. The authors concluded that CRYO may be a useful adjunct to TXA to improve survival in patients requiring blood transfusion. No data on the rate of thromboembolic complications was reported.

Although most studies find that TXA confers a mortality benefit among trauma patients, there are some studies with discordant findings. A 2014 case control study by Valle et al. found that among 300 patients who required emergency surgery and/or transfusions, mortality was higher among the TXA group compared to the control group (27% vs 17%, *p* = 0.024) [8]. Similarly, a 2015 retrospective review by Harvin et al. found that among 1,032 patients with hyperfibrinolysis, in-hospital mortality was higher in the TXA group than the control group (40% vs. 17%, *p* < 0.001) [7].

### Pre-hospital TXA use

As the above studies found TXA to be a safe and effective drug to reduce in-hospital bleeding and mortality after major trauma, subsequent works investigated the potential utility of TXA in the pre-hospital setting. In 2020, Guyette et al. published results from The Study of Tranexamic Acid During Air Medical and Ground Pre-hospital Transport (STAAMP) randomized clinical trial [6]. In this study, there was no significant difference in mortality at 30 days between the TXA and placebo groups (8.1% versus 9.9%, *p* = 0.17). However, in subgroup analysis, there was a mortality benefit among patients who received TXA within 1 h of injury compared to those that received placebo (4.6% vs. 7.6%, *p* < 0.001). In addition, patients with severe shock who received TXA had a lower 30-day mortality compared with the placebo group (18.5% vs. 35.5%, *p* < 0.003). There was no significant difference in the incidence of PE, DVT, or seizures across groups. These results are in line with prior studies demonstrating that the benefit of TXA is greatest when administered shortly after injury and in patients with more severe injuries. Similarly, the 2023 multi-center PATCH RCT evaluated the effects of pre-hospital TXA administration on survival [37]. The author concluded that mortality at 24 h was lower among patients receiving TXA compared to placebo (9.7% vs. 14.1%, RR = 0.69, 95% CI 0.51–0.94), but survival outcomes at 6 months was not significantly different between the two groups (53.7% in the TXA group vs. 53.5% in the placebo group, RR = 1.00, *p* = 0.95). The number of serious adverse events, including thromboembolic complications, did not differ significantly between the groups.

### TXA use in orthopaedic and spine trauma

Orthopaedic surgery after extremity trauma often results in acute blood loss with associated need for blood transfusions. Following the publication of the CRASH-2 trial, orthopaedic traumatologists became interested in using this drug to reduce blood loss during surgery. Many studies have been completed examining the safety and efficacy of TXA for reducing blood loss after proximal femur and pelvis/acetabulum fractures. Fewer studies exist that examine the use of TXA for fractures of the distal femur, tibia, foot/ankle, and upper extremity. This may be because these injuries are less common than hip fractures and are associated with lower blood loss than pelvis/acetabulum fractures. Compared to general surgery literature, orthopaedic literature is more likely to report the effect of TXA on blood loss as opposed to mortality, as the baseline mortality rate is very low following isolated orthopaedic injuries. Key studies are summarized in Table 2.

**Table 2:** Key studies on the use of TXA in orthopaedic and spine trauma contexts

Author name, year	Study design	Sample size	Key findings on TXA efficacy	Key findings on TXA safety
Agius et al. [31]	Meta-analysis	1,194 hip fractures	Pooled analysis showed that patients in the TXA group had significantly lower transfusion requirements compared to the no-TXA group (RR 0.50, *p* = 0.009)	There were no statistically significant differences in the rate of thromboembolic events (RR 0.01, 95% CI − 0.02 to 0.04, P = 0.47)
Bloom et al. [44]	Meta-analysis	1,834 hip fractures	Average total blood loss was 258 mL lower for patients who received high-dose TXA compared to controls (*p* = 0.042) and 223 mL lower for patients who received low-dose TXA compared to controls (*p* < 0.001)	There was no statistically significant difference in rates of thromboembolic complications between the control group, low dose TXA group, and high dose TXA group
Kim et al. [32]	Meta-analysis	764 pelvis and acetabulum fractures	Pooled analysis showed no significant difference in estimated blood loss (mean difference = − 64.67, *p* = 0.29) or transfusion rate (OR 0.77, *p* = 0.71) between the TXA and no-TXA groups	There was no significant difference in the rate of venous thromboembolism (OR 1.53, *p* = 0.50) or postoperative infection (OR 1.15, *p* = 0.90)
Sen et al. [33]	Randomized controlled trial	63 acetabulum fractures	There was no significant difference in quantity of intraoperative blood loss (438.11 ml vs. 442.81, *p* = 0.947) or rate of blood transfusion (8 patients vs. 5 patients, *p* = 0.719) between the groups	No patient in either group had a thromboembolic event
Tan et al. 2021	Randomized controlled trial	150 ankle fractures	TXA groups had significantly lower total blood loss and the effect was more pronounced with higher dose (placebo TBL = 382 mL, low dose TBL = 282 mL, medium dose TBL = 246 mL, high dose TBL = 198 mL, and ultra-high dose TBL = 176 mL, *p* < 0.001)	No patients experienced a symptomatic venous thromboembolism
Tang et al. [46]	Meta-analysis	255 calcaneus fractures	The TXA group had significantly lower blood loss over the first 24 h compared to the no-TXA group (Standard mean difference = − 0.99, *p* < 0.001). However, there was no significant difference after 24 h	There was insufficient data to calculate differences in the rate of thromboembolic complications
Cuff et al. [47]	Randomized controlled trial	101 proximal humerus fractures	The TXA group had less average intraoperative blood loss (178 mL vs. 129 mL, *p* < 0.0001 and less total blood loss (280 mL vs. 188 mL, *p* < 0 .0001) compared to the no-TXA group	There were no thromboembolic complications in either group
Vasu et al. [34]	Meta-analysis	828 thoracolumbar fractures	The TXA group had lower total blood loss (standard mean difference = − 2.54, *p* = 0.0001) and intraoperative blood loss (standard mean difference = − 0.96, *p* < 0.00001) compared to the no-TXA group	There were no significant differences in rates of thromboembolic events

Hip fractures, including femoral neck and intertrochanteric fractures, have been studied most extensively. A 2022 meta-analysis by Agius et al. included 13 trials and 1,194 patients with femoral neck or intertrochanteric fractures [31]. Pooled results from the meta-analysis demonstrated that patients who received TXA had significantly lower relative risk of transfusion (RR = 0.50, *p* = 0.009) compared to patients who did not receive TXA. There was no statistically significant difference in the rate of thromboembolic complications (RR = 0.01, *p* = 0.47). Similarly, a 2023 meta-analysis by Bloom et al. performed a network analysis using data from 19 RCTs including 1,890 patients with femoral neck or intertrochanteric fractures [44]. The authors found that TXA significantly decreased total blood loss (TBL) and that the effect was more pronounced at higher doses compared to lower doses. Average TBL was 258 mL lower for patients who received high-dose TXA compared to controls (*p* = 0.042) and 223 mL lower for patients who received low-dose TXA compared to controls (*p* < 0.001). There was no statistically significant difference in rates of thromboembolic complications.

Pelvis and acetabulum fractures are less common than intertrochanteric or femoral neck fractures. However, they are often associated with significant blood loss and increased morbidity. A large meta-analysis by Kim et al. [32] found no difference in operative blood loss or thromboembolic complications with TXA use during pelvis and acetabular fracture surgeries. Similarly, a randomized control trial by Sen et al. that included 63 acetabulum fractures found no significant difference in the amount of intraoperative blood loss (438.11 mL vs. 442.81, *p* = 0.947) or rate of blood transfusion (8 patients vs. 5 patients, *p* = 0.719) between the TXA and no-TXA groups [33]. There were no thromboembolic complications in either group [33]. These results suggest unclear benefit of TXA administration for pelvis and acetabulum fractures.

Blood loss during surgery for fractures of the foot and ankle is typically lower than blood loss during surgery for fractures of the proximal femur and pelvis; however, TXA may still be a useful tool in these scenarios. In a study looking at TXA use for ankle fracture surgery, Tan et al. found that IV TXA significantly decreased TBL and that the effect was more pronounced with higher dosages of TXA (placebo TBL = 382 mL, low dose TBL = 282 mL, medium dose TBL = 246 mL, high dose TBL = 198 mL, and ultra-high dose TBL = 176 mL, *p* < 0.001) [45]. No patient in any group experienced a symptomatic venous thromboembolism. Similarly, in a meta-analysis examining TXA use during fixation of calcaneus fractures, Tang et al. found that TXA significantly reduced TBL over the first 24 h (Standard mean difference = − 0.99, *p* < 0.001) [46]. However, there was no significant effect of TXA on TBL beyond 24 h (no *p*-value reported). The included studies administered TXA via topical or IV formulations at a variety of doses. The meta-analysis had insufficient data to calculate differences in the rate of thromboembolic complications.

Limited studies exist that examine the use of TXA after fractures of the upper extremity; however, there is some data on TXA use after proximal humerus fractures. Cuff et al. performed an RCT of 101 patients who received either a single preoperative dose of 1 g intravenous TXA or a matched control [47]. They found that the TXA group had less average intraoperative blood loss (178 mL vs. 129 mL, *p* < 0.0001), and lower TBL (280 mL versus 188 mL, *p* < 0.0001). There were no thromboembolic complications in either group. Little has been published on the use of TXA for fractures of the elbow and forearm; however, hemodynamically significant blood loss from these injuries is rare, and therefore TXA may be less necessary for these injuries.

Spine surgery in the setting of trauma is also associated with significant perioperative blood loss, and although multiple recent studies support the use of TXA in elective spine surgery [48, 49], literature on TXA use for perioperative spinal trauma is more limited. Nearly all existing literature focuses on thoracolumbar fractures. Research on TXA use for cervical spine surgery consists primarily of surgeries for compressive myelopathies [50]. In a 2022 meta-analysis, Vasu et al. provides a comprehensive review of existing literature on TXA use after thoracolumbar trauma and found that both total blood loss and intraoperative blood loss was significantly lower for patients that received TXA [total blood loss standard mean difference (SMD) =  − 2.54, *p* = 0.0001] [intraoperative blood loss SMD =  − 0.96, *p* < 0.00001] [34]. There were no significant differences in transfusion requirements, operative duration, deep vein thrombosis, or pulmonary embolism.

In conclusion, there is a significant body of literature detailing the efficacy and safety of TXA in orthopaedic trauma and spine surgery. Current evidence suggests that TXA can help reduce bleeding during hip fracture surgery [31, 44], though it may have less benefit during surgeries for fractures of the pelvis/acetabulum [32, 33]. More limited evidence exists regarding the use of TXA for fractures of the distal femur, tibia, foot/ankle, and upper extremity; however, some studies have documented potential benefit after ankle and proximal humerus fractures [45, 47]. Limited studies are available regarding TXA use after spine trauma; however, the present findings are promising and should encourage ongoing investigation in this area. Regardless of injury type, TXA is a very safe drug, with all studies documenting no increased risk of thromboembolic complications after receiving TXA [31–33, 45–47].

### TXA use in neurotrauma

Intracranial hemorrhage is a common complication following TBI and is associated with significant morbidity and mortality [51]. TXA has been hypothesized to mitigate secondary brain injury following the initial TBI insult, though study results on the benefit of TXA are mixed. Key studies are summarized in Table 3.

**Table 3:** Key studies on the use of TXA in neurotrauma contexts

Author name, year	Study design	Sample size	Key findings on TXA efficacy	Key findings on TXA safety
CRASH-2 Collaborators [52]	Randomized controlled trial	270	Mean total hemorrhage growth was lower in the TXA group, but this did not achieve statistical significance (adjusted difference = − 3.8 mL, *p* = 0.33). There was a trend towards decreased mortality in the TXA group (adjusted OR 0.47, *p* = 0.06) that did not achieve statistical significance	There authors reported no adverse events regarded as serious, unexpected, or suspected to be related to the study treatment. Data on thromboembolic complications was not reported
CRASH-3 Trial Collaborators [36]	Randomized controlled trial	12,737	There was no significant difference in the risk of head injury-related death between the TXA (18.5%) and control (19.8%) groups (RR = 0.94, 95% CI 0.86–1.02). However, in the subgroup analysis, there was a significantly lower risk of head-injury related death among patients with mild-to-moderate head injury who were treated with TXA compared to the control group (RR = 0.79, 95% CI 0.64–0.95)	The risk of DVT/PE was not significantly different between the TXA and placebo groups (RR = 0.98, 95% CI 0.74–1.28). Risk of seizures was also not significantly different between groups (RR = 1.09, 95% CI 0.90–1.33)
Rowell et al. 2022	Randomized controlled trial	966	There was no significant difference in favorable neurologic function at 6 months between the TXA group (65%) and the placebo group (62%) (*p* = 0.84). There was also no statistically significant difference in mortality at 28 days (14% in the TXA group vs. 17% in the placebo group, *p* = 0.26) or progression of intracranial hemorrhage (16% in the TXA group vs. 20% in the placebo group, *p* = 0.16)	The risk of DVT/PE was not significantly different between the TXA and placebo groups (RR = 0.98, 95% CI 0.74–1.28). Risk of seizures was also not significantly different between groups (RR = 1.09, 95% CI 0.90–1.33)
Bossers et al. [54]	Randomized controlled trial	1,827	In the unadjusted analysis, the TXA group had significantly higher 30-day mortality (OR 1.34, *p* < 0.001) compared to the control group, but after adjusting for confounding variables, including injury severity, there was no significant difference (OR 1.17, *p* = 0.35). However, on subgroup analysis, there were substantially higher odds of death within 30 days for patients with severe isolated TBI who received pre-hospital TXA compared to similar patients who did not receive TXA (OR 2.05, *p* = 0.007)	There was no data reported on thromboembolic events

The earliest data on TXA use for traumatic brain injury came from the CRASH-2 Intracranial Bleeding Study in 2011. This was a nested prospective RCT using data from the CRASH-2 trial to evaluate the effect of early administration of TXA on intracranial hemorrhage in patients with TBI [52]. In total, 270 patients were randomized to either a 1 g loading dose of TXA followed by a 1 g infusion over 8 h or a placebo. While mean total hemorrhage growth was lower in the TXA group, this did not achieve statistical significance (adjusted difference = − 3.8 mL, *p* = 0.33). Similarly, the authors reported a trend towards decreased mortality in the TXA group (adjusted OR 0.47, *p* = 0.06).

In 2019, the CRASH-3 randomized controlled trial was published, building upon the results of CRASH-2. CRASH-3 randomized over 12,000 TBI patients to receive either a 1 g IV TXA bolus followed by a 1 g TXA infusion over 8 h or a matched placebo [36]. There was no significant difference in the risk of head injury-related death between the TXA (18.5%) and control (19.8%) groups (RR = 0.94, 95% CI^1^ 0.86–1.02*). However, in the subgroup analysis, there was a significantly lower risk of head-injury related death among patients with mild-to-moderate head injury, defined as GCS between 9 and 15, who were treated with TXA compared to the control group (RR = 0.79, 95% CI 0.64–0.95). This difference was not observed in patients with severe head injury, defined as GCS < 9 (RR = 0.99, 95% CI 0.91–1.07). The risk of DVT/PE was not significantly different between the TXA and placebo groups (RR = 0.98, 95% CI 0.74–1.28).

Similarly, Rowell et al. conducted a large pre-hospital multicenter RCT evaluating the impact of early TXA administration on neurological outcomes at six months for patients with head trauma [53]. There was no significant difference in favorable neurologic function at 6 months between the TXA groups (65%) and the placebo group (62%) (*p* = 0.84). There was also no statistically significant difference in mortality at 28 days (14% in the TXA groups vs. 17% in the placebo group, *p* = 0.26) or progression of intracranial hemorrhage (16% in the TXA groups vs. 20% in the placebo group, *p* = 0.16). No statistical analysis of thromboembolic events was performed.

An additional large multi-center study on TXA after TBI was published in 2021 by Bossers et al. This study, titled the Brain Injury: Pre-hospital Registry of Outcome, Treatments and Epidemiology of Cerebral Trauma (BRAIN-PROTECT) study, included 1,827 patients with suspected severe TBI based on trauma mechanism, clinical findings, and GCS < 8 [54]. The study included 693 who received TXA and 1,134 who did not receive TXA. Most patients in the TXA group received a 1 g IV bolus in the pre-hospital setting. Both an unadjusted logistic regression model, which did not account for confounders, and a multivariable logistic regression model, which did account for cofounders, were constructed to model 30-day mortality. In the unadjusted analysis, the TXA group had significantly higher 30-day mortality (OR 1.34, *p* < 0.001) compared to the control group, but after adjusting for confounding variables, including injury severity, there was no significant difference (OR 1.17, *p* = 0.35). However, on subgroup analysis, there were substantially higher odds of death within 30 days for patients with severe isolated TBI who received pre-hospital TXA compared to similar patients who did not receive TXA (OR 2.05, *p* = 0.007). This study did not report on thromboembolic events.

Overall, results on the use of TXA after TBI are mixed. While some studies show that TXA reduces the volume of intracranial hemorrhage for select patients with TBI [55], others do not find a statistically significant impact [52, 53]. Multiple large, well-designed studies have found that TXA does not significantly reduce mortality after TBI [52–55]. TXA seems to be more beneficial for patients with mild-to-moderate TBI than severe TBI [36, 54]. TXA is not associated with increased risk of DVT/PE when administered for TBI [36, 55].

### Strengths and limitations

In this narrative review, we summarize the existing body of evidence regarding the efficacy, safety, and cost of TXA across multiple trauma contexts. This review benefited from the inclusion of studies from multiple fields, a focus on key trials, and the inclusion of studies with conflicting results. While the narrative design of this review allowed the authors to provide a broad summary of TXA use in multiple trauma contexts, there is a risk of selection bias. We mitigated this risk by requiring multiple authors to review the final selection of included studies. The heterogeneity of included studies is another potential weakness. While we sought to include only studies of the highest methodological quality, there is variability in the quality of published literature between different surgical fields. Finally, as this is a narrative review, it lacks the rigor of a systematic review. We attempted to partially mitigate this by prioritizing transparency regarding the methodology.

## Conclusions

Though some studies suggest limited or no mortality benefit of TXA, particularly in the prehospital setting [6–8], multiple large, high-quality clinical trials have found that TXA significantly reduces mortality among trauma patients. Most notably, the CRASH-2 trial found that empiric TXA administration resulted in a 1.5% absolute reduction in 28-day mortality among patients with or at risk for hemodynamically significant bleeding [5]. Current literature also supports the use of TXA after orthopaedic trauma [31, 36, 44]. Extensive evidence exists that TXA is a safe medication, with numerous studies demonstrating no increased risk of thromboembolic events after administration of TXA in trauma settings [5, 6, 30–34, 36, 37, 41, 45, 47, 55]. Future research on TXA is needed to elucidate the potential benefit of TXA after traumatic brain and spine injury and the optimal dose and route of administration of TXA.

## Data Availability

No datasets were generated or analysed during the current study.

## Abbreviations

TXA: Tranexamic acid
RCT: Randomized clinical trial
IV: Intravenous
IM: Intramuscular
FDA: Food and Drug Administration
DVT: Deep vein thrombosis
WHO: World Health Organization
LYs: Life years
UK: United Kingdom
QALY: Quality-adjusted life years
ISS: Injury severity score
CRYO: Cryoprecipitate
RR: Relative risk
TBL: Total blood loss
SMD: Standard mean difference
OR: Odds ratio

## Footnotes

1 *This study reports confidence intervals rather than *p*-values.
